# Fabrication of Polyacrylonitrile UF Membranes by VIPS Method with Acetone as Co-Solvent

**DOI:** 10.3390/membranes12050523

**Published:** 2022-05-15

**Authors:** Alexey Yushkin, Alexey Balynin, Mikhail Efimov, Konstantin Pochivalov, Inna Petrova, Alexey Volkov

**Affiliations:** 1 A.V. Topchiev Institute of Petrochemical Synthesis RAS, 29 Leninsky Prospekt, 119991 Moscow, Russia; ab@ips.ac.ru (A.B.); efimov@ips.ac.ru (M.E.); pkv@isc-ras.ru (K.P.); ivpetrova@ips.ac.ru (I.P.); avolkov@ips.ac.ru (A.V.); 2 G.A. Krestov Institute of Solution Chemistry of the Russian Academy of Sciences, 1 ul. Akademicheskaja, 153045 Ivanovo, Russia

**Keywords:** polyacrylonitrile, acetone, co-solvent, VIPS, pore size, MWCO, membrane, ultrafiltration

## Abstract

For the first time, a systematic study was carried out of the replacement of the low-volatility solvents N-methyl-2-pyrrolidone (NMP) or dimethylsulfoxide (DMSO) with the high-volatility solvent acetone in the casting solution of polyacrylonitrile (PAN). The effect of acetone’s presence in the casting solution on the performance of ultrafiltration membranes fabricated via vapor-induced phase separation (VIPS) was investigated. It was possible to replace 40% of NMP and 50% of DMSO with acetone, which resulted in the reduction of the casting solution viscosity from 70.6 down to 41.3 Pa∙s (20% PAN, NMP), and from 68.3 down to 20.6 Pa∙s (20% PAN, DMSO). It was found that 20 min of exposure to water vapor (relative humidity—85%) was sufficient to govern the phase separation, which was mainly induced by the water vapor. Regardless of the casting solution composition (15 or 20% PAN; DMSO or NMP), all membranes formed via VIPS possessed a sponge-like porous structure. The addition of acetone to the casting solution allowed the reduction of the transport pore size from 35–48 down to 8.5–25.6, depending on the casting solution composition. By varying the acetone content at constant polymer concentration, it was possible to decrease the molecular weight cut-off (MWCO) from 69 to 10 kg/mol. Membranes prepared from 20% PAN solution in an acetone/DMSO mixture had the lowest MWCO of 10 kg/mol with a water permeance of 5.1 L/(m^2^·h·bar).

## 1. Introduction

Polymeric membranes have a wide range of applications in the separation of liquids and gases due to their usability, ecological compatibility, and low cost [1,2,3]. Membranes with various pore sizes have been developed, depending on the requirements of different applications. There are a few polymeric materials for ultrafiltration and microfiltration membrane preparation, namely polysulfone (PSF) [4,5], polyethersulfone (PES) [6], cellulose acetate (CA) [7], poly(vinylidene fluoride) (PVDF) [8], polypropylene (PP) [9,10], polyacrylonitrile (PAN) [11,12,13], and others [14,15].

PAN is a material that is widely used for the fabrication of micro- and ultrafiltration membranes for aqueous [12,13] and nonaqueous applications [13,16] due to its good mechanical and film-forming properties and stability in solvents like hydrocarbons, alcohols, or mild aprotic solvents (e.g., dichloromethane, acetone) [17,18,19]. The stability of PAN results from the intermolecular dipole–dipole interactions of the nitrile side groups and the absence of functional groups in the polymer backbone [20]. Most commercial membranes have an asymmetric porous structure, fabricated via the phase inversion process wherein a polymer solution is cast on a support, followed by immersion in a nonsolvent bath where polymer precipitation and membrane formation take place [3,21]. For PAN membrane preparation, the casting solution is obtained by the dissolution of a polymer in aprotic solvents N,N-dimethylformamide (DMF), N-methyl-2-pyrrolidone (NMP), or dimethylsulfoxide (DMSO). The limited number of suitable solvents restricts the possible pore size of the obtained membranes. As a result, it is very difficult to reduce the pore size of the PAN membrane.

The surface pore size and porosity of a membrane depend on the relative diffusion rate of solvent and precipitant [21]. Casting solution composition, casting conditions, and coagulation bath composition affect phase inversion parameters. There are different ways to regulate membrane structure and performance by changing the compositions of the casting solution and coagulation bath, for instance, the addition of secondary components into the casting solution [21]. In the case of PAN, polyethylene glycol (PEG) [22,23], polyvinyl pyrrolidone (PVP) [24,25], or salts [26,27] are usually used for membrane preparation.

On the other hand, for the preparation of nanofiltration membranes, volatile solvents such as acetone, 1,4-dioxane, or tetrahydrofuran (THF) are often added into the casting solution [28]. It is believed that the partial evaporation process of the volatile co-solvent before the immersion precipitation step in the coagulation bath is necessary for the formation of a top layer. The molecular weight cutoff and mean pore size of the membranes decrease with increasing solvent evaporation time [28]. It can be supposed that the evaporation of the volatile co-solvent leads to an increase in polymer concentration and hence a decrease in pore size and porosity [29].

Solvent mobility also impacts the pore structure of the obtained membranes. If solvent outflow from the casting solution is faster than nonsolvent inflow, a membrane with a smaller pore size and a denser skin layer is formed [21]. In the case of PAN solutions with a volatile component, the casting process involves the displacement of a mild precipitant (for instance acetone) with a hard precipitant (water).

A theoretical investigation of the phase inversion process requires consideration of both thermodynamic and kinetic aspects of the system. The most useful instrument for the description of a three-component system comprising polymer, solvent, and nonsolvent is a ternary phase diagram [30,31,32,33]. For polymer/solvent/nonsolvent systems, the position of the binodal can be found. The pathway of solution composition, which depends on formation conditions, determines the phase inversion parameters. At the same time, the description of thermodynamic characteristics for quaternary polymer solutions consisting of a polymer and three low-molecular-weight liquids is still a difficult task. Hansen solubility parameters are often applied to characterize the polymer–solvent interactions [34]

The square of the solubility parameter of a given component, which is equal to the cohesive energy density, is divided into three contributions: *δ*^2^ *= δ_d_*^2^ *+ δ_p_*^2^ *+ δ_h_*^2^, where *δ_d_*, *δ_p_* and *δ_h_* are the dispersive, the polar, and the hydrogen-bonding terms. The higher the thermodynamic compatibility of the polymer (*P*) and solvent (*S*), the smaller the distance *R_P-S_* between them in the Hansen space:

(1) $$R_{P-S}=\sqrt{4\cdot {\left(\delta_{d,P}-\delta_{d,S}\right)}^{2}+{\left(\delta_{p,P}-\delta_{p,S}\right)}^{2}+{\left(\delta_{h,P}-\delta_{h,S}\right)}^{2}}$$

If this distance is less than the so-called interaction radius of the polymer *R*0 (radius of the sphere of solubility in the Hansen space), which determines the boundary between good and poor solvents, the polymer must dissolve in this solvent. For PAN *R*0 = 10.9 MPa^1/2^ [13,34]. The *R_P-S_* for good PAN solvents is 7.1–10.2 MPa^1/2^ (Table 1). On the other hand, the common volatile co-solvents acetone, 1,4-dioxane, and THF are located in Hansen space further from PAN than the radius of solubility, and hence they are poor solvents. This limits the use of these liquids for the dissolution of PAN. Nevertheless, a limited amount of these liquids still can be added to the casting solution.

The purpose of this work was to investigate the effect of addition of acetone to the casting solution in PAN membrane preparation. The influence of acetone on pore size and filtration performance was demonstrated. Acetone was chosen as a volatile component because it has a lower molecular weight than THF and 1,4-dioxane and hence a higher diffusivity.

## 2. Materials and Methods

### 2.1. Materials

Acrylonitrile monomer (>99.5) was purchased from Fluka (Switzerland). Dimethyl sulfoxide, DMSO (>99%), NMP (>99%), DMF (>99%), acetone (>99%), ammonium peroxodisulfate, sodium dithionite, sulfuric acid, potassium carbonate, lysozyme (MW = 14.4 kg/mol), pepsin (34.5 kg/mol), ovalbumin (45 kg/mol), and bovine serum albumin (BSA, MW = 69 kg/mol) were acquired from Khimmed (Russia). Helium with a purity of at least 99.95% was purchased from the Moscow Gas Refinery Plant.

### 2.2. PAN Synthesis

The synthesis of PAN was carried out in an aqueous medium in the presence of a redox system consisting of ammonium peroxodisulfate ((NH_4_)_2_S_2_O_8_) and sodium dithionite (Na_2_S_2_O_4_) as initiators. First, 300 mL of bidistilled water was added into an Erlenmeyer flask. Next, sulfuric acid and monomer were added consequentially with concentrations [H_2_SO_4_] = 1.9 × 10^−2^ mol/L and [acrylonitrile] = 1.27 mol/L. The initiators were added to the flask simultaneously with concentrations [(NH_4_)_2_S_2_O_8_] = 5.88 × 10^−3^ mol/L, [Na_2_S_2_O_4_] = 2.52 × 10^−3^ mol/L. The prepared solution was shaken and placed in a thermostat for 40 min at 60 °C. A 100 mL solution of sulfuric acid [H_2_SO_4_] = 1.9 × 10^–2^ mol/L and monomer [acrylonitrile] = 0.66 mol/L was added and the reaction continued for 4 h. The polymer was then filtered, washed sequentially in a solution of potassium carbonate in water and methanol to remove sulfuric acid, and then dried in vacuo to a constant weight. The polymer yield was 92.4%.

The study of the molecular weight characteristics of the resulting polymer was carried out using gel permeation chromatography (GPC) on a GPC-120 chromatograph (PolymerLabs). The analysis was carried out at 50 °C in DMF. The average molecular weight of the synthesized PAN (M_w_) was 118,800 g/mol. The obtained polymer was characterized by a polydispersity index (M_w_/M_n_) of 3.2.

### 2.3. Solution Preparation and Characterization

Various polymeric solutions containing different concentrations of PAN, solvent, and acetone were prepared in this work. Solvent (NMP or DMSO) was mixed with acetone in the weight ratio of 90/10, 80/20, 70/30, 60/40, or 50/50 and then PAN was added to the solution. The PAN content in the resulting solutions was 15 or 20 wt.%. The addition of components was carried out at a temperature of 20 °C and humidity of 20%. The flask with the mixture of components was hermetically sealed to prevent the evaporation of acetone. The solution was treated in an ultrasonic bath for 30 min with further stirring for 7 days at 45 °C to produce a homogeneous solution. The obtained solution was treated in an ultrasonic bath again for 30 min to complete homogenization and remove air bubbles. The prepared solution was stored in darkness in a hermetically sealed flask at 20 °C and 20% humidity. The resulting compositions of the casting solution are presented in Table 2.

The dynamic viscosity of the prepared solutions was measured at 20 °C using a Brookfield DV2T-RV viscometer.

Three-component phase diagrams of the PAN/solvent/acetone and PAN/solvent/water systems were obtained at 25 °C by determining cloud points. PAN solutions of various concentrations were prepared in containers and titrated under constant stirring at 25 °C. The titrant, which was in the burette, was added dropwise until the stirred solution became cloudy. The cloud point was fixed when the cloudiness of the polymer solution did not disappear within 24 h [35,36].

### 2.4. Membrane Preparation

Using the vapor-induced phase separation (VIPS) method, the cast film was placed in a sealed box with constant relative humidity (RH) of 85%. The obtained membrane was then washed for 5 min in distilled water to remove the solvent. After completion of the phase separation, all membranes were washed with distilled water and then kept in fresh distilled water for 24 h to wash out the solvent residues. The water was then replaced with fresh water for further storage of the obtained membranes until use.

### 2.5. Membrane Characterization

Filtration experiments were carried out in a dead-end stirred filtration cell. The membrane samples were placed on porous stainless steel disks and sealed with rubber O-rings. The active membrane area was 7.9 cm^2^. At least three coupons were measured from every membrane, and the permeance data were averaged. The difference in permeance measured for different membrane coupons was up to 10%. The system was pressurized with helium. Membrane permeance was measured from the beginning of the experiment without initial prepressurization. Permeate samples were taken every 5 min until constant permeance was obtained, and then five more measurements were taken. Sample permeance was determined as the average of the last five data points. In the case of solution filtration, the feed was stirred at 550 rpm to minimize the concentration polarization effect. The membrane permeance *L_P_* (L/(m^2^·h·bar)) was determined as follows:

(2) LP=JΔp=m(ρ·S·t·Δp),

where *J* is the flux of the liquid, Δ*p* is the transmembrane pressure (bar), *m* is the mass of the permeate (g), *ρ* is the density of the permeate (g/L), *S* is the active membrane area (m^2^), and *t* is the filtration time (h). The transmembrane pressure was 5 bar.

The following solutes were selected to characterize the separation performance of the membranes: PEG, lysozyme, pepsin, ovalbumin, and BSA. PEG had molecular weights of 1, 2, 5, and 10 kg/mol. A new membrane coupon was used for every rejection experiment, which were carried out at the transmembrane pressure of 5 bar for at least 2 h to achieve steady-state values of rejection. Before the rejection test, distilled water was filtered through the membrane coupon for 1 h at 5 bar. Phosphate buffer (0.1 M) with a pH of 7.0 was used for BSA solution preparation. Other proteins and PEG were dissolved in distilled water. All solutions were prepared with a solute concentration of 0.5 g/L. The concentration of proteins in the feed and permeate was measured with a spectrophotometer at the wavelength of 280 nm. PEG content was determined via the weight method after the evaporation of solvent at 80 °C to a constant weight. The rejection *R* was calculated using the following relationship:

(3) R=(1−CpCf)·100%,

where *C_f_* and *C_p_* denote the solute concentrations in the feed and permeate, respectively.

The pore size was measured via liquid–liquid displacement porosimetry (LLDP) [13,37] using the porometer POROLIQ 1000 ML (Porometer, Belgium). The operating principle is based on the measurement of the equilibrium pressure corresponding to the flux of the displacing liquid. The displacement of the wetting liquid was carried out by a stepwise increase of the transmembrane pressure with monitoring of the flux through the membrane after 180 s initial stabilization time at each applied pressure. The measurement was stopped after a linear dependence of the flux on pressure was reached, which indicated a complete displacement of the wetting liquid. The alcohol-rich phase was used as the wetting liquid and the water-rich phase was used as the displacing liquid. Three coupons (2 cm in diameter) were cut from every membrane and were placed into the beaker with the wetting liquid for at least 2 h at 20 °C before testing. The results were averaged for all investigated samples. The measurements were carried out at 25 °C using a pair of immiscible liquids prepared by demixing a mixture of isobutanol and water (1/4, *v/v*). The diameter (*D*) of the open pore is related to the transmembrane pressure via the Young–Laplace equation:

(4) D=4·γ·cosθΔp,

where *γ* is the interfacial tension between the two liquids, *θ* is the contact angle between the membrane and the wetting liquid (complete wetting is assumed, i.e., cos *θ* = 1), and Δ*p* is the transmembrane pressure. Interfacial tension *γ* for the mixture of isobutanol and water is 1.9 mN/m at 25 °C. Pore size was calculated according to the procedure described in detail in [13].

The porosity of the membranes was determined by the sorption of water into the dry sample. For this purpose, membrane coupons with measured dimensions (length, width, and thickness) and weight were immersed in water for 24 h. The typical size of coupons in this experiment was 4–5 cm and the thickness was 80–140 µm. After 24 h of soaking, the coupon was taken out of the water and excess liquid was removed from the membrane surface with filter paper. The sample weight was then measured with an analytical balance. Membrane porosity (*ε*) was determined according to the following equation:

(5) $$\varepsilon =\frac{m_{wet}-m_{dry}}{\rho_{water}\cdot V_{sample}}\cdot 100\%,$$

where *m_wet_* and *m_dry_* are the membrane weights before and after immersion in water, consequently, *ρ_water_* is the water density, and *V_sample_* is the sample volume.

Scanning electron microscopy (SEM) was used to characterize the structure and morphology of the membranes. SEM was carried out on a Thermo Fisher Phenom XL G2 Desktop SEM (Waltham, MA, USA). Cross-sections of the membranes were obtained using liquid nitrogen after preliminary impregnation of the specimens in isopropanol. A thin (5–10 nm) gold layer was deposited on the prepared samples in a vacuum chamber (~0.01 mbar) using a Cressington 108 auto Sputter Coater desktop magnetron sputter (Watford, UK). The accelerating voltage used during image acquisition was 15 keV.

## 3. Results and Discussion

### 3.1. Characterization of PAN/DMSO/Acetone and PAN/NMP/Acetone Mixtures

**Figure 1:**
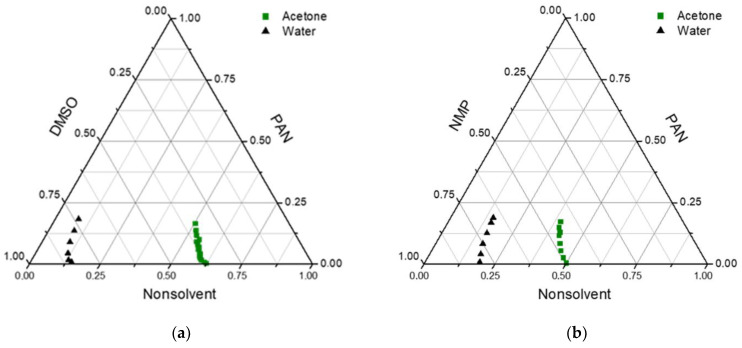
Ternary phase diagrams for (**a**) PAN/DMSO/acetone and PAN/DMSO/water mixtures; (**b**) PAN/NMP/acetone and PAN/NMP/water mixtures.

In Figure 1, the experimental cloud point data for PAN in different solvent/nonsolvent mixtures are presented. As can be seen, the concentration of acetone in the PAN solution could be higher than 50% without the appearance of turbidity. Two regions appeared in the diagrams, one-phase and two-phase regions. The two-phase part consists of the metastable and unstable regions separated by the spinodal curve. Spinodal decomposition occurs when the composition reaches an unstable region, leading to liquid–liquid demixing. In these cases, bigger pores and higher porosity should be obtained. When the composition stays in the metastable region, phase separation happens via the nucleation and growth mechanism [38]. This makes the membrane structure denser and decreases pore size.

In the obtained diagrams, the miscibility region was considerable for water and was much bigger for acetone. Due to low precipitant inflow in the VIPS method, acetone evaporation also increased the duration of the composition path in the metastable region. This means that nucleation and growth mechanisms were more probable in membranes obtained in this way. Because of the low molecular weight of acetone, their outflow in the membrane preparation process was high, leading to an increase in polymer concentration and a denser selective layer on the membrane surface.

On the other hand, water is a much harder precipitator than acetone, and a lower amount of water led to phase separation. This means that if acetone fully evaporates from a polymer solution in the evaporation step of membrane preparation, the PAN concentration can be increased up to 2–2.5 times and phase separation occurs in regions inaccessible without the addition of acetone.

**Figure 2:**
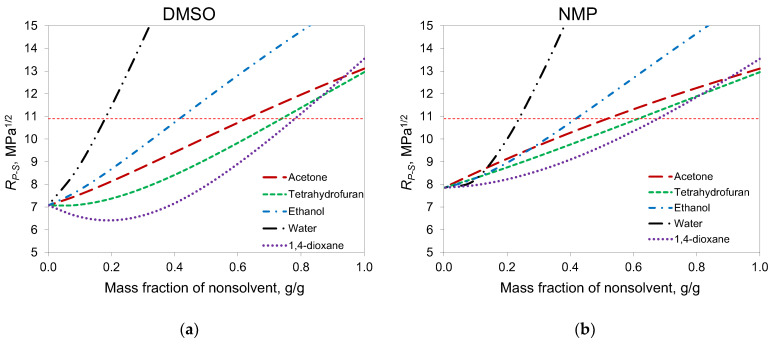
Distance in Hansen space between PAN and binary mixtures of nonsolvents with (**a**) DMSO and (**b**) NMP. Horizontal dashed line—PAN solubility radius.

According to the phase diagrams, in the case of DMSO, acetone can be added to a solution with a higher concentration than in the case of NMP. This can be explained by the fact that, according to Hansen solubility parameters, DMSO is a better solvent for PAN than NMP (Table 1). The calculation of distance in Hansen space between PAN and a binary solvent consisting of two liquids, calculated according to [39], gave a good correlation with the cloud point data (Figure 2). For NMP, R_P-S_ became higher than the PAN solubility radius when the mass fraction of acetone was higher than 0.5, and cloud points were obtained at the same concentrations. For DMSO, this concentration was 0.65.

The calculation of Hansen distances for other common co-solvents, namely 1,4-dioxane and THF, showed that these solvents can be added in greater proportions than acetone. At the same time, acetone is more interesting for membrane formation due to its lower viscosity and molecular weight.

PAN solutions in mixtures of NMP and acetone were prepared with ratios of 90/10, 80/20, 70/30, and 60/40. We failed to prepare a solution with a 50/50 ratio because dissolution was too slow for this mixture and acetone evaporation became significant. In the case of DMSO, the highest achieved acetone/solvent ratio was 50/50.

**Figure 3:**
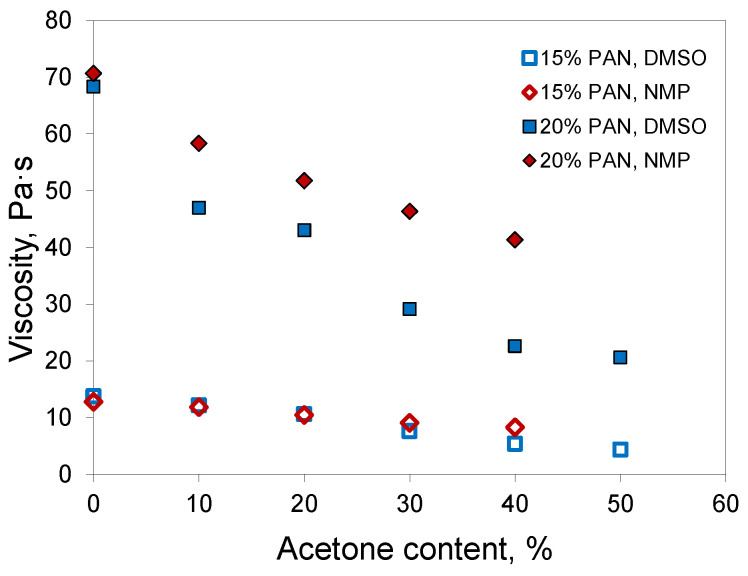
Dynamic viscosity of PAN solutions in solvent/acetone binary mixtures.

Experimental measurements of the dynamic viscosity of PAN solutions in mixtures with acetone demonstrated that the addition of acetone decreased the viscosity of the polymer solution (Figure 3). In the case of DMSO, the drop in viscosity was more distinct, which could be associated with the higher intrinsic viscosity of DMSO. The addition of a low-viscosity component had a more significant effect on the viscosity of the resulting fluid.

Hence, acetone decreases the viscosity of PAN solutions, which is useful for the optimization of casting solution viscosity in hollow-fiber preparation because it promotes a further increase of polymer concentration in the casting solution when the desired polymer concentration cannot be achieved with a common single solvent.

### 3.2. Effect of Vapor Exposure Time on Membrane Properties

**Figure 4:**
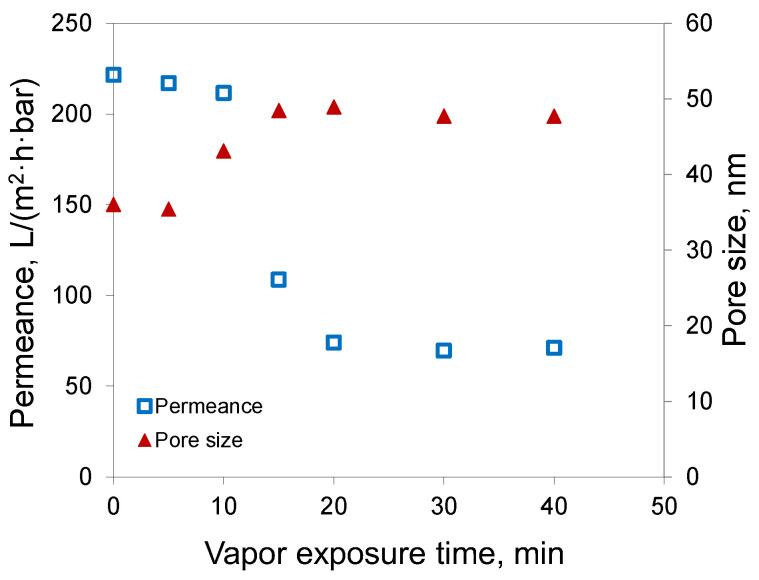
Water permeance and transport pore size of resulted membranes as function of exposure time to the air at RH 85% prior to immersion in the water bath (casting solution: 15% PAN, DMSO).

In the first stage of membrane preparation, the vapor exposure time of polymeric solution to the humid air (RH = 85%) before the washing in a water bath was varied to identify the optimal conditions for membrane formation using the VIPS method. Figure 4 shows the effect of the duration of water vapor exposure of the casting solution on the water permeance and transport pore size of the membranes prepared from 15% PAN solution in DMSO.

It can be seen that there were three different regions, which can be attributed to the different factors that led to the phase separation of the polymer solution. The short contact of the polymer solution with the water vapors (up to 10 min) was not sufficient to drive the polymer solution out of equilibrium, and the phase separation was triggered by the contact with the nonsolvent (water) in the washing bath. In this case, the phase inversion proceeded by a mechanism typical for NIPS. As the result, the membranes showed nearly the same water permeance of 210–220 L/(m^2^∙h∙bar) at 0–10 min.

With a long exposure time, the membranes demonstrated water permeances of 70–74 L/(m^2^∙h∙bar), although the exposure time was increased from 20 up to 40 min. From this, it can be concluded that the membrane was formed during the contact of the polymeric solution with water vapor before immersion in the water bath (VIPS). In between, the porous structure of the membranes was formed by the combination of NIPS/VIPS.

It is interesting to note that the results for the membrane pore size (see Figure 4) demonstrated that the water vapor started to affect membrane morphology at the contact time of 10 min, with an increase of pore size from 35 up to 43 nm; meanwhile, the membranes demonstrated about the same pore size of 48–49 nm at the exposure time of 15 min or greater.

**Figure 5:**
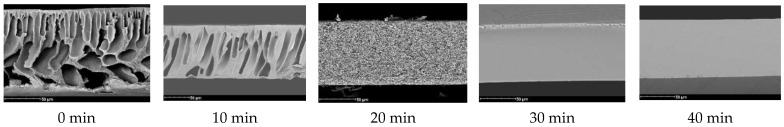
SEM visualization of the membrane cross-section with respect to vapor exposure time in the air at a relative humidity of 85% before immersion in the water bath (casting solution: 15% PAN, DMSO).

The analysis of membrane cross-sections visualized by SEM revealed that the membranes possessed a finger-like porous structure at the time of 10 min; meanwhile, all the membranes showed a sponge-like structure when the exposure time was 20 min or more (see Figure 5). Bearing this in mind, the exposure time of 20 min at an RH of 85% was selected for the fabrication of all membranes in this work.

To study the evaporation rate of acetone, the solutions of PAN/acetone/solvent with higher acetone contents (40 or 50%) were deposited on glass with a previously measured weight. The thickness of the polymer film was 200 µm. The glass with the solution was then placed on an analytical balance, and the dependence of the weight on time was recorded. The measurements were carried out at an air humidity of 20% and a temperature of 20 °C. To calculate acetone evaporation, it was assumed that there was no solvent evaporation or water absorption. It was found that 90% of the acetone contained in the polymer solution evaporated within 4–8 min. It means that by the time the phase separation begins, the main part of acetone has time to evaporate.

### 3.3. Separation Performance of the Membranes Prepared by VIPS Method

The smallest pore size was obtained from the solutions with the highest acetone concentration (Table 3). An increase in acetone content monotonically decreased membrane pore size and permeance. In the case of NMP, the acetone/solvent ratio of 40/60 yielded a membrane with pore sizes of 25.6 nm and 11.8 nm for 15 and 20% PAN content, respectively. The permeances of these membranes were 44 and 14.6 L/(m^2^·h·bar). In the case of 50/50 DMSO/acetone mixture, the permeances were 17.2 and 5.1 L/(m^2^·h·bar) and the pore sizes were 15.6 and 8.5 nm for 15 and 20% PAN content, respectively.

**Figure 6:**
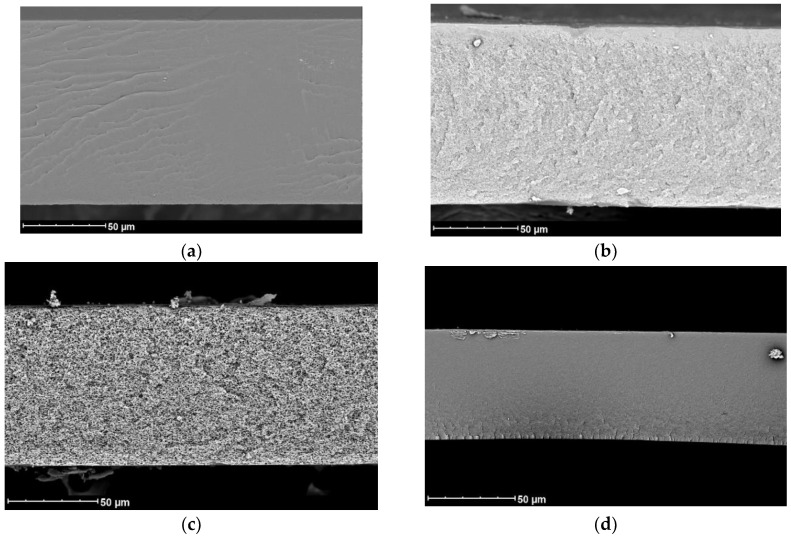
SEM images of membranes prepared via VIPS method from 15% PAN solutions in (**a**) NMP; (**b**) acetone/NMP 40/60; (**c**) DMSO; (**d**) acetone/DMSO 50/50.

**Figure 7:**
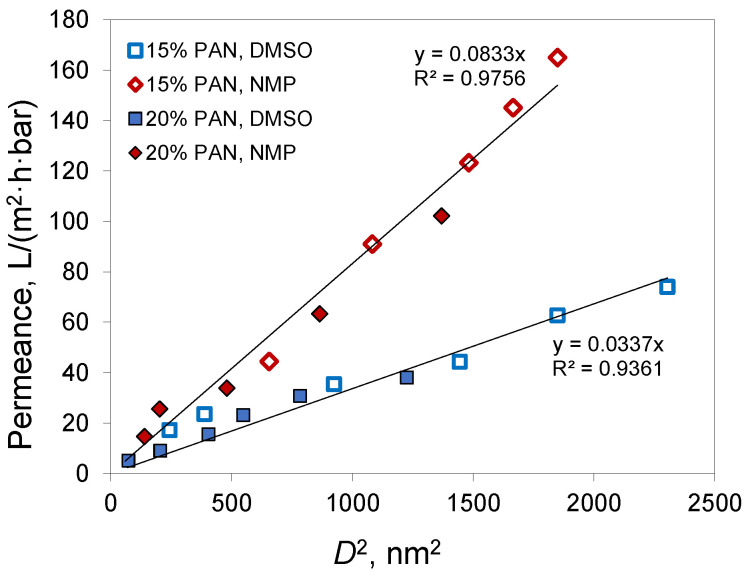
The plot of water permeance versus pore diameter (*D*) squared for membranes prepared using VIPS method.

Unlike NIPS, membranes prepared using the VIPS method do not have finger-like macrovoids and a spongy structure usually forms. Depending on the preparation conditions, membranes obtained using the VIPS method can have a bicontinuous, cellular, or nodular structure [40]. SEM images confirmed the absence of macrovoids in the membranes obtained via the VIPS method (Figure 6). For the membranes prepared from a 15% PAN solution in DMSO, a cellular structure was observed, while for other membranes SEM resolution did not allow the membrane structure to be distinguished. At the same time, the plot of membrane permeance versus pore diameter (D) squared (Figure 7) showed a linear dependence for both solvents used for membrane preparation.

**Figure 8:**
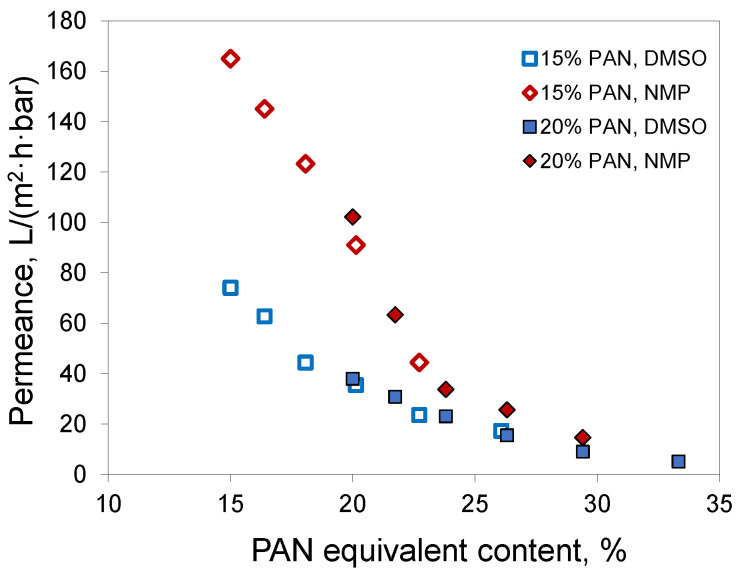
Plot of water permeance versus PAN equivalent content for different PAN/NMP/acetone and PAN/DMSO/acetone casting solutions.

Since acetone mostly evaporates from the polymer film before precipitation, the phase separation follows the same path as if a polymer solution without acetone, but with a higher PAN concentration, was employed to prepare the membrane. For brevity, we denote this concentration as PAN equivalent content. This is not the PAN concentration at the precipitation moment, since it does not consider water uptake and assumes full acetone evaporation. In the case of a 20% PAN solution in DMSO/acetone 50/50, the equivalent content was 33%. For 15% PAN solutions at the acetone/solvent ratio of 30/70, the equivalent content was nearly 20%. If full acetone evaporation takes place before phase separation starts, then the characteristics of the membranes should correspond to those of membranes obtained from solutions with the same PAN equivalent content. A comparison of membranes obtained from solutions of 15% PAN in DMSO/acetone 70/30 and NMP/acetone 70/30 with the corresponding membranes from 20% PAN solutions in DMSO and NMP without acetone confirmed this assumption (Figure 8). The membranes obtained using the same solvent and similar PAN equivalent content had similar permeances. In the case of DMSO, the membranes had a permeability of 35 and 38 L/(m^2^·h·bar) at a pore size of 30 and 35 nm, respectively. In the case of NMP, similar parameters were 91 and 102 L/(m^2^·h·bar) with a pore size of 33 and 37 nm. Apparently, the tendency for the membranes with acetone addition to have slightly smaller pore size and permeance was due to the fact that not all the acetone evaporated.

Increasing the polymer concentration in the casting solution is a logical way to obtain denser membranes with smaller pore sizes. On the other hand, this increase is associated with an increase in solution viscosity, which limits the polymer concentration. The application of volatile co-solvents and their evaporation before the immersion precipitation step overcomes this limitation. The investigation of membrane preparation via the VIPS method carried out in this work confirmed that this also works in the case of PAN, although acetone is a poor solvent for this polymer. For instance, in the case of a 20% PAN solution and DMSO, the presence of acetone in the casting solution decreased pore size from 35 to 8.5 nm (4.1 times) (Table 3). Other combinations provided a less dramatic effect: 2.1 and 1.6 times for 15% and NMP, 3 times for 15% and DMSO.

From Figure 7 and Table 3 it can be concluded that NMP produced membranes with a smaller pore size and higher permeance in comparison to DMSO. This can be explained by the higher pore connectivity or porosity in membranes prepared from NMP. At the same time, the membrane porosity is determined by the amount of polymer in the casting solution and the contraction of the film during phase inversion. The obtained membrane porosity was close for both solvents at similar casting solution compositions (Table 3). Hence, the difference in the porosity of the selective layer cannot explain the difference in permeance for membranes from different solvents.

Thus, decreasing membrane permeance with an increase of polymer content was connected with a decrease in membrane porosity, while the difference between membranes prepared from NMP and DMSO probably resulted from different pore connectivity.

**Figure 9:**
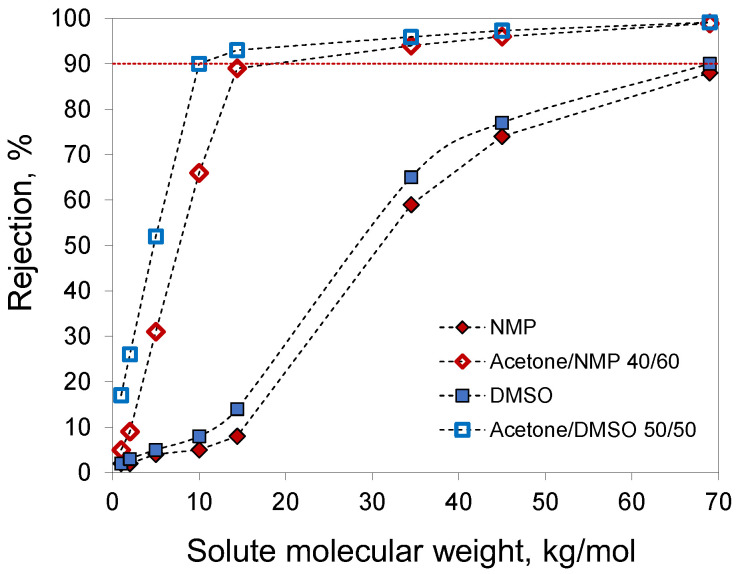
Molecular weight cut-off curves obtained with membranes prepared from 20% PAN solutions in NMP, acetone/NMP 40/60, DMSO, and acetone/DMSO 50/50.

For membranes prepared from 20% PAN solutions in NMP, acetone/NMP 40/60, DMSO, and acetone/DMSO 50/50, the molecular weight cut-off (MWCO) was determined using solutes with different molecular weights in water (Figure 9). Membranes prepared with the addition of acetone demonstrated higher rejections due to the smaller pore size. The extrapolation of the obtained data to a rejection of 90% gave an MWCO of 10 kg/mol for the membrane prepared from a 20% PAN solution in acetone/DMSO 50/50. The other studied membranes had MWCO values of 18.4 and 69 kg/mol for acetone/NMP 40/60 and DMSO, respectively. For membranes from 20% PAN solution in NMP, MWCO values were not determined due to lower rejection.

The obtained membranes demonstrated lower permeance in comparison to membranes prepared using the NIPS method. For instance, in [12], membranes demonstrated permeance of 500–1500 L/(m^2^·h·bar) but the concentration of PAN in the casting solution was 8–13%. Membranes prepared via the NIPS method from 15% PAN solution in DMSO had a permeance of 150 L/(m^2^·h·bar) [13], which is two times higher than the membrane permeance obtained in this work using the VIPS method at the same composition. In this work, the membrane obtained via the NIPS method from 15% PAN solution in DMSO demonstrated a permeance of 220 L/(m^2^·h·bar) (Figure 4, the point at zero vapor exposure time). The difference in membrane permeance obtained via NIPS in this work and in [13] can be explained by the difference in molecular weight of the polymer and hence in solution viscosity. On the other hand, membranes prepared by thermally induced phase separation and by a combined method of phase separation induced by contact of hot casting solution and cold precipitant in some cases demonstrated permeance less than 50 L/(m^2^·h·bar) despite the micron pore size [23]. This difference can be attributed to the different structures of the obtained membranes and the different pore connectivity. The spongy structure without macrovoids is considered more desirable since it possesses a less defective membrane surface and higher tensile stretch in comparison to a finger-like porous structure. This can be useful for membrane supports in thin-film membrane preparation. In the case of NIPS, a spongy structure of PAN membranes can be obtained by the addition of a pore-forming agent (e.g., glycerol, PVP, PEG) to the casting solution. The high viscosity of PVP and PEG limits polymer concentration in the casting solution. At the same time, the permeance of membranes with a spongy structure obtained via NIPS method by addition of PVP was 50–150 L/(m^2^·h·bar) [41]. This is comparable to the permeance obtained in this work despite the lower polymer concentration in the casting solution. Thus, the combination of high-volatility co-solvent with the VIPS method can be considered a complementary approach to the NIPS method when the fabrication of ultrafiltration membranes made of PAN with a sponge-like porous structure is desired.

## 4. Conclusions

In this work, it was shown that low-volatility solvents such as NMP or DMSO in a polymer casting solution can be partially replaced with high-volatility acetone, which brings additional benefits to the fabrication of porous PAN membranes via the VIPS method due to faster evaporation of the solvent. Additionally, the change of polymer and/or acetone concentration and the nature of the low-volatility solvent (NMP or DMSO) in the casting solution provide certain flexibility in control of the porous structure and filtration performance of the resulted UF membranes with sponge-like morphology. For instance, it was shown that the replacement of up to 40 or 50% of NMP or DMSO in the casting solution with acetone allowed variance, depending on PAN concentration, of the water permeance from 5.1 up to 165 L/(m^2^∙h∙bar), and the mean size of transport pores from 8.5 up to 48 nm. The tightest MWCO of 10 kDa was obtained for the membranes fabricated from 20% PAN solution in acetone/DMSO (50/50); the water permeance was 5.1 L/(m^2^∙h∙bar).

Although the VIPS method requires the additional step of prolonged exposure of the casting solution to water vapor before membrane washing, the formed membranes mostly possessed a sponge-like porous structure even in the case of high polymer concentration in the casting solution (e.g., 20%). In the more widely used and traditional NIPS method, PAN membranes possessed a finger-like porous structure, while sponge-like structures could be realized at lower PAN concentrations (<15–16%) by the addition of pore-forming agents (e.g., glycerol, PVP, PEG) to the casting solution. Thus, the combination of high-volatility co-solvents with the VIPS method can be considered a complementary approach to the NIPS method when the fabrication of ultrafiltration membranes made of PAN with a sponge-like porous structure is desired.

## Figures and Tables

**Table 1:** Hansen solubility parameters (from [34]), viscosity, and molecular weight of good and poor PAN solvents according to the distance in Hansen space (*R_P-S_*).

	Material	*η*	*Mw*	*δ_t_*	*δ_d_*	*δ_p_*	*δ_h_*	*R_P-S_*
		mPa·s	g/mol	MPa^1/2^	MPa^1/2^	MPa^1/2^	MPa^1/2^	MPa^1/2^
	PAN	-	-	27.4	21.7	14.1	9.1	-
good solvents	DMSO	1.80	78	26.7	18.4	16.4	10.2	7.1
	NMP	1.53	99	24.0	18.0	12.3	7.2	7.8
	DMF	0.77	73	24.9	17.4	13.7	11.3	8.9
	DMAc	0.88	87	22.8	16.8	11.5	10.2	10.2
Non solvents	THF	0.48	72	19.5	16.8	5.7	8	13.0
	Acetone	0.32	58	19.9	15.5	10.4	7	13.1
	1,4-dioxane	1.31	88	20.5	19.0	1.8	7.4	13.5
	Ethanol	1.19	46	26.5	15.8	8.8	19.4	26.5
	Water	1.00	18	47.8	15.5	16.0	42.3	35.5

*η*—Viscosity.

**Table 2:** Casting solution compositions.

Solvent	Acetone/Solvent Ratio	Component Content in the Casting Solution, wt.%		
		PAN	Solvent	Acetone
DMSO	0/100	15	85	0
	10/90		76.5	8.5
	20/80		68	17
	30/70		59.5	25.5
	40/60		51	34
	50/50		42.5	42.5
	0/100	20	80	0
	10/90		72	8
	20/80		64	16
	30/70		56	24
	40/60		48	32
	50/50		40	40
NMP	0/100	15	85	0
	10/90		76.5	8.5
	20/80		68	17
	30/70		59.5	25.5
	40/60		51	34
	0/100	20	80	0
	10/90		72	8
	20/80		64	16
	30/70		56	24
	40/60		48	32

**Table 3:** Membrane permeance and pore sizes for membranes prepared from different solutions.

PAN Content	Solvent	Acetone/Solvent Ratio	Pore Size, nm	Porosity, %	Permeance, L/(m^2^·h·bar)
15	DMSO	0/100	48	76	74
		10/90	43	69	63
		20/80	38	63	44
		30/70	30	58	35
		40/60	19.7	51	23.5
		50/50	15.6	47	17.2
15	NMP	0/100	43	75	165
		10/90	41	67	145
		20/80	38	62	123
		30/70	33	55	91
		40/60	25.6	48	44
20	DMSO	0/100	35	57	38
		10/90	28	51	31
		20/80	23.4	46	23.1
		30/70	20.1	42	15.5
		40/60	14.3	37	9.0
		50/50	8.5	32	5.1
20	NMP	0/100	37	59	102
		10/90	29	54	63
		20/80	21.9	47	34
		30/70	14.2	41	26
		40/60	11.8	35	14.6

## Data Availability

The data presented in this study are available on request from the corresponding author. The data are not publicly available due to restrictions on the open data publication by the organization where the study was performed.
